# A genome-wide scan for genes under balancing selection in the plant pathogen *Ralstonia solanacearum*

**DOI:** 10.1186/s12862-019-1456-6

**Published:** 2019-06-17

**Authors:** José A. Castillo, Spiros N. Agathos

**Affiliations:** 1 School of Biological Sciences and Engineering, Yachay Tech University, Hacienda San Jose s/n and Proyecto Yachay, Urcuquí, Ecuador; 2 School of Biological Sciences and Engineering, Yachay Tech University, Hacienda San Jose s/n and Proyecto Yachay, Urcuquí, Ecuador

**Keywords:** Balancing selection, Ralstonia solanacearum, Tajima’s D, Watterson’s theta, Fu & Li’s D*, Virulence related genes, Pathogenesis

## Abstract

**Background:**

Plant pathogens are under significant selective pressure by the plant host. Consequently, they are expected to have adapted to this condition or contribute to evading plant defenses. In order to acquire long-term fitness, plant bacterial pathogens are usually forced to maintain advantageous genetic diversity in populations. This strategy ensures that different alleles in the pathogen’s gene pool are maintained in a population at frequencies larger than expected under neutral evolution. This selective process, known as balancing selection, is the subject of this work in the context of a common bacterial phytopathogen. We performed a genome-wide scan of *Ralstonia solanacearum* species complex, an aggressive plant bacterial pathogen that shows broad host range and causes a devastating disease called ‘bacterial wilt’.

**Results:**

Using a sliding window approach, we analyzed 57 genomes from three phylotypes of the *R. solanacearum* species complex to detect signatures of balancing selection. A total of 161 windows showed extreme values in three summary statistics of population genetics: Tajima’s D, θ_w_ and Fu & Li’s D*. We discarded any confounding effects due to demographic events by means of coalescent simulations of genetic data. The prospective windows correspond to 78 genes with known function that map in any of the two main replicons (1.7% of total number of genes). The candidate genes under balancing selection are related to primary metabolism and other basal activities (51.3%) or directly associated to virulence (48.7%), the latter being involved in key functions targeted to dismantle plant defenses or to participate in critical stages in the pathogenic process.

**Conclusions:**

We identified various genes under balancing selection that play a significant role in basic metabolism as well as in virulence of the *R. solanacearum* species complex. These genes are useful to understand and monitor the evolution of bacterial pathogen populations and emerge as potential candidates for future treatments to induce specific plant immune responses.

**Electronic supplementary material:**

The online version of this article (10.1186/s12862-019-1456-6) contains supplementary material, which is available to authorized users.

## Background

Balancing selection (BS) is a well-known concept in evolutionary biology and population genetics that has been extensively analyzed in many organisms. BS is a type of positive selection that favors the maintenance of a high genetic diversity within a given population. This diversity could be displayed as an excess of polymorphisms on existing alleles or as the maintenance of different alleles at selected loci. Usually BS influences genetic variation in genomes in a localized way, maintaining diversity at the selected sites but also increasing diversity at closely linked neutral sites [1]. BS works through different mechanisms, namely, heterozygote advantage (also called overdominant selection, [2]), frequency-dependent selection [3] and spatial/temporal heterogeneity [4]. One particularly interesting case is frequency-dependent selection that is related to the coevolution between host and pathogen following the ‘trench warfare’ model. This model postulates that coevolution of both host and pathogen leads to stable richness of polymorphisms through BS [5]. Good examples of this model are interactions of plant resistance genes with virulence-related genes of the pathogen under defined ecological and epidemiological conditions specific for each host-pathogen system. In this case, elevated polymorphism levels in virulence genes have been found in several pathogens and therefore reflect an arms race behavior between host and pathogen. However, not only pathogens show an increased level of diversity, but also the plant hosts, since they have to defend themselves from a broad arsenal of virulence molecules from the pathogens. This leads to a complex interplay between plants and pathogens consisting of antagonistic coevolution that promotes diversity in both partners; here, diversifying and balancing selection interact to create and maintain the genetic diversity [5]. This plant-microbe interaction seems to be very ancient, as many defense pathways of non-flowering and flowering plants existed in the last common land plant ancestor [6].

Lately, much attention has been paid to BS in different eukaryotic species such as humans [7, 8], plants [9] and parasites [10], however, very little to bacteria, with few exceptions [11–15]. At the level of plant bacterial pathogens, a couple of articles report BS in *Pseudomonas viridiflava* [13] and *P. syringae* [14]. These two works analyze particular genome regions that show BS signatures. In this work, we focus on performing a genome-wide scan to detect genes under BS in the plant bacterial pathogen *Ralstonia solanacearum*.

*R. solanacearum* belongs to the Betaproteobacteria class and the Burkholderiaceae family and is considered a species complex (RSSC) because it is composed of a number of genetic groups, often subdivisible into several different monophyletic lineages called phylotypes [16, 17]. Four phylotypes have been recognized in RSSC, each one reflecting a distinct geographic origin: phylotype I (Asia), phylotype II (Americas), phylotype III (Africa), and phylotype IV (Indonesia) [17, 18]. Phylogenetic studies show that phylotype II is also divided into two monophyletic subgroups designated as IIA and IIB [18]. RSSC has lately been re-classified in three different species: the species name ‘*R. solanacearum*’ remains for phylotype II, whereas strains of phylotype IV, *Ralstonia syzygii* and blood disease bacterium (BDB), the causal agent of the banana blood disease are reclassified inside the species *R. syzygii*, and finally, phylotypes I and III form a new single bacterial species designated as *R. pseudosolanacearum* [19, 20].

Strains belonging to the RSSC are aggressive plant pathogens that cause wilt disease of more than 250 plant species including economically valuable crops. These bacteria alternate between two lifestyles, as saprophytic on soil and water, and as pathogenic inside plant tissues and organs. The bacteria enter susceptible plants through the roots, invade the xylem vessels, form biofilms and spread to the aerial parts of the plants. For pathogenesis, RSSC strains use an ample repertoire of molecular weapons like cell wall degrading enzymes, an extracellular polysaccharide (EPS) and effectors secreted through the type III secretion system (T3SS) [21]. All virulence factors are expressed and eventually secreted in a coordinated manner and appear to have additive effects since no single factor can completely explain infection and disease symptoms [22]. At the genomic level, the RSSC strains harbor two DNA circular molecules, a large replicon of 3.7 Mb and a smaller 2.1 Mb replicon, corresponding to chromosome and megaplasmid respectively. Both replicons contain housekeeping as well as virulence-related genes [23].

To investigate BS in the RSSC, we performed a genome-wide scan on both replicons (chromosome and megaplasmid) and attempted to determine whether BS is more frequent in essential versus virulence-related genes. Only for the purposes of this work, we have considered each RSSC phylotype (including subgroups IIA and IIB) as single, independent populations and have measured the excess of common polymorphisms using the classical summary statistics (Tajima’s D and others; [24]) rather than rely on model-based methods [25] or new summary statistics (like β, [26]) because it was considered that they would not add more confidence to the results when used together with Tajima’s D.

## Results

### Genome sequence alignment and population parameters

For all analyses performed in this work, we chose to work with locally collinear blocks (LCBs) than with complete genome alignments because LCBs produce aligned and concatenated sequences composed of homologous regions of sequence shared by the genomes under study. In this way, only conserved segments that appear to be internally free from genome rearrangements were considered for population parameters and summary statistics calculations. This is critical for calculations aimed at detecting polymorphisms on aligned sequences. Genome alignments of the RSSC phylotypes analyzed in this work produced a variable number of LCBs that concatenated represent about (or higher than) 50% of their respective genomes (except for the megaplasmid of phylotype IV, see Table 1). Because some genome sequences from the database are poor in megaplasmid sequences, we were only able to align seven genome sequences for the phylotype IIA megaplasmid (Table 1).

**Table 1:** RSSC genomic sequences used in this analysis and population parameters and summary statistics calculated for whole replicons sequence data

Phylotype/replicon	Number of genomes analyzed	Number of nucleotides analyzed	Percentaje of GMI1000 replicon^*a*^	ρ (per site)^*b*^	θ (per site) ^*b*^	ρ/θ	Tajima’s D	θ_w_ (per site)	Fu-Li’s D*
I/chromosome	20	1,907,685	51.33	0.0120	0.0052	2.399	−0.438	0.0051	−0.676
I/megaplasmid	20	1,282,321	61.22	0.0233	0.0067	3.470	−0.450	0.0067	−0.743
IIA/chromosome	12	1,971,855	53.06	0.0008	0.0103	0.071	1.084	0.0101	0.895
IIA/megaplasmid	7	938,400	44.80	0.0020	0.0164	0.125	−0.642	0.0160	−0.725
IIB/chromosome	20	1,451,109	39.05	0.0007	0.0092	0.071	0.923	0.0080	0.539
IIB/megaplasmid	20	927,177	44.27	0.0010	0.0133	0.075	0.930	0.0116	0.522
IV/chromosome	5	1,957,952	52.68	0.0011	0.0112	0.088	−0.434	0.0104	−0.467
IV/megaplasmid	5	503,195	24.02	0.0021	0.0139	0.136	−0.420	0.0134	−0.468

^*a*^ As a reference, the GMI1000 chromosome has 3,716,413 bp and the megaplasmid 2,094,509 bp [23]

^*b*^ ρ and θ: per site recombination and mutation rate, respectively

Alignments were analyzed for information on population parameters, which are necessary for the demographic simulations (see below). Per site recombination rate (ρ) and per site mutation rate (θ) vary across different phylogenetic groups in RSSC (Table 1). The chromosome of phylotype IIB and the chromosome of phylotype I show the lowest value for ρ and θ respectively. On the other hand, the highest values of the two parameters are shared by the megaplasmid of phylotype I (for ρ) and the megaplasmid of phylotype IIA (for θ). Interestingly, the relation ρ/θ gives opposite values depending on the phylotype. Phylotypes II (A and B) and IV show values lower than 1 for ρ/θ, while phylotype I reaches values higher than that. This result suggests that the role played by recombination seems to be uneven across RSSC lineages and that recombination had a stronger influence on introducing nucleotide substitution relative to mutation in phylotype I (both replicons) than in other phylotypes.

### Summary statistics

**Fig. 1:**
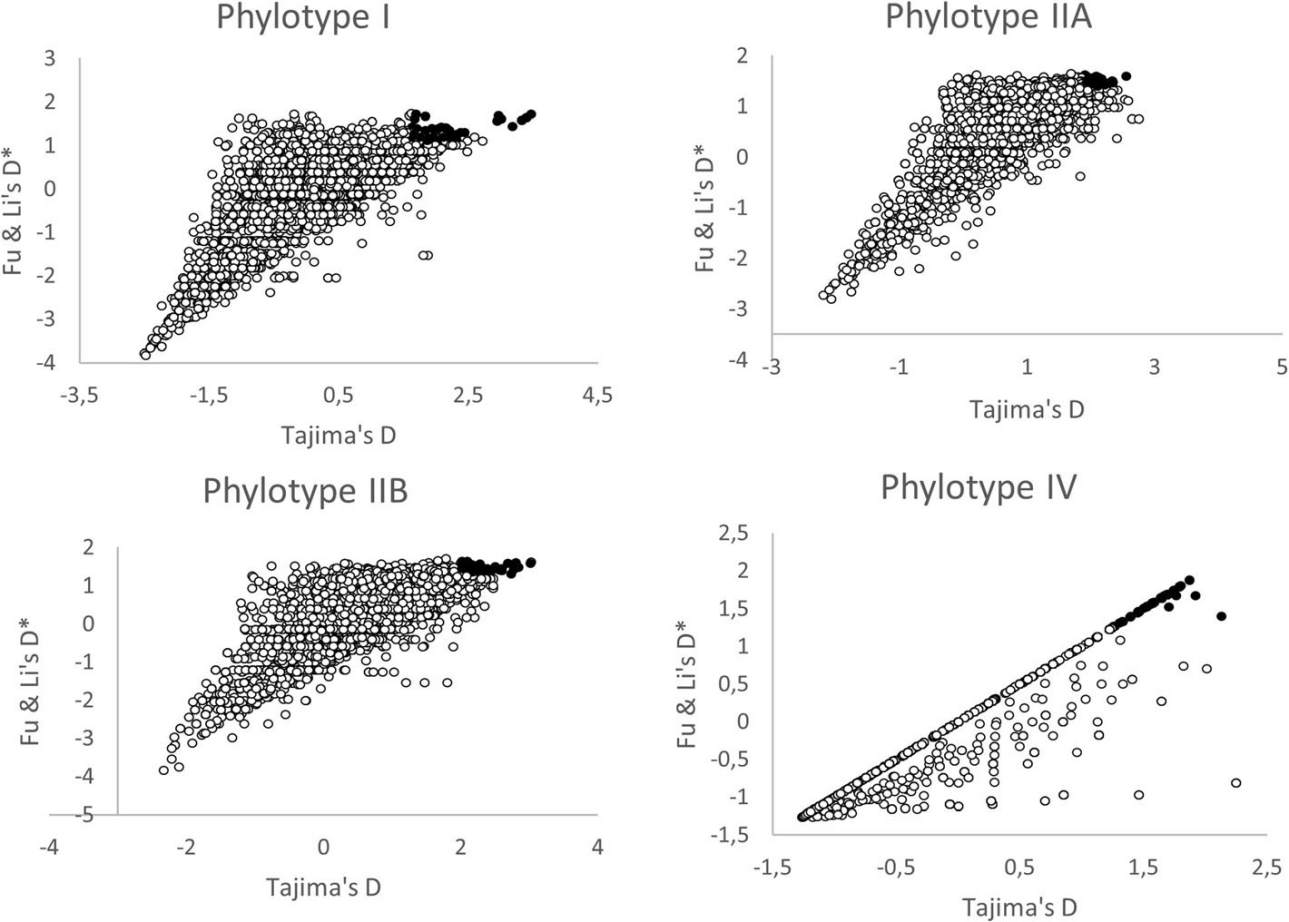
Two-dimensional plot of Tajima’s D and Fu & Li’s D* values for all windows. Shaded dots represent the 5% top windows of the distribution of both measures of BS

RSSC genome alignments were scanned for BS signatures in both replicons (i.e. chromosome and megaplasmid). As stated above, RSSC is classified into four phylotypes. We focused the analysis on phylotype I, II and IV as there were not enough genome sequences available in the databases for phylotype III at the time of the analysis and phylotype II was analyzed in both its subclusters (IIA and IIB), as they were separate and independent phylogenetic groups (IIA and IIB, Table 1). The extent of polymorphism was measured by using the three summary statistics (θ_w_, Tajima’s D, and Fu & Li’s D*). The Tajima’s D test is useful to analyze whether a DNA sequence is evolving different from neutrality, therefore, it helps to detect selection. The Tajima’s D values calculated for the whole replicon of each phylotype ranged from − 0,6,417,465 to 1084 depending on phylotype (Table 1). Phylotypes I and IV show Tajima’s D distribution shifted towards negative values in both replicons, as well as phylotype IIA (megaplasmid). On the contrary, phylotypes IIA (chromosome) and IIB (both replicons) show a tendency towards positive values. Fu & Li’s D* results follow a similar pattern as Tajima’s D. This suggests that both these summary statistics are highly correlated, an aspect that is confirmed later (see below). When we estimated the summary statistics using the sliding window strategy, different values for the three statistics were obtained when calculated for each phylotype and replicon. After eliminating windows without SNPs, we observed extreme Tajima’s D values (such as 3.46 and − 2.506 for the chromosome in phylotype I) but also moderate values, along all windows analyzed (Additional file 1: Table S1). The tendency towards negative values was also reflected in Tajima’s D and Fu & Li’s D* mean values of sliding windows analysis for phylotypes I (both replicons), IIA (megaplasmid) and IV (both replicons) (Additional file 1: Table S1, Fig. 1). Watterson’s θ values are relatively high for all phylotypes except for phylotype I and IIB (chromosome). The slight differences between θ and θ_w_ observed in Table 1 are due to the way this statistic is calculated, as in one case we employed the Bayesian method and in the other the formula proposed by Watterson [27].

A two-dimensional plot of all three summary statistics suggests that their values are correlated (Fig. 1). To confirm a possible correlation between them, we calculated the Spearman rank correlation coefficient between θ_w_, Tajima’s D and Fu & Li’s D* using the sliding window data. As expected, results show that there is a strong pairwise correlation among the three summary statistics for all phylotypes and replicons except for phylotype I when comparing θ_w_ and Tajima’s D (Additional file 1: Table S2). In some cases, a very high positive correlation was observed, as is the case of phylotypes IIA and IV for Tajima’s D-Fu & Li’s D* combination (0.738, 0.964 and 0.982, 0.976, respectively) suggesting a strong agreement between these statistics. This result also supports the idea that the high values of the correlation among the summary statistics point out to real BS signatures (or demographic structuring) in the aligned sequences rather than being random values.

### Simulations and candidate genes under balancing selection

We tested whether the unusual incidence of high values of summary statistics obtained from aligned sequences was due to BS on RSSC genomes or reflected effects of demography. We adopted the widely used strategy based on simulation of genetic data under the coalescent framework. Three most plausible demographic scenarios were tested as null models: standard neutral model (SNM), population contraction model (PCM) and bottleneck model (BNM). SNM assumes the population does not change in net size (constant size) and is under neutrality, PCM indicates the population of constant size has contracted to a smaller size and BNM, assumes the population has reduced to a smaller size, for a number of generations and afterward, the population has suddenly recovered its original size [28]. Although these models may not represent the exact history of RSSC populations because of their intrinsic complexity, this approximation is sufficiently advantageous to be used as a set of null demographic models focused upon reducing false positives. We included in our analysis the gene cluster *agr* from *S. aureus* as a positive control [11]. We analyzed 3537 bp of the *agr* cluster using the standard procedure for BS signature detection in RSSC aligned sequences as detailed in the Data and Methods section. This analysis produced 18 windows, however, in none of them, we obtained maximum matching values for the three summary statistics. As expected, windows with high observed values of Tajima’s D, θ_w_, or Fu & Li’s D* showed very significant values after simulations according to the demographic models tested in this work (observed values: Tajima’s D = 2.72677**; θ_w_ = 0.05249**; Fu & Li’s D* = 1.73125**, the double asterisk meaning significant difference at *p* < 0.05 compared to values obtained with simulations for SNM). After having demonstrated confidence in the analysis using this positive control, we applied the same procedure to scan the RSSC aligned sequences. Results show (Table 1) that the top 5% of the distribution of the summary statistics exceed the respective simulated values (under the corresponding demographic model) in most of the cases, as validated by hypothesis testing significance. Note that the power of this detection resides in the concurrent consideration of all three statistics, Tajima’s D, θ_w_, and Fu & Li’s D*. This result provides a robust evidence that the windows with high values of summary statistics correspond to genes or genomic regions under BS (Table 1). Subsequently, we identified the genes overlapping the candidate windows (Table 2). A list of unidentified genes (unknown gene function) or windows corresponding to intergenic regions is detailed in Additional file 1: Table S3.

**Table 2:** Identity and probable function of genes showing highest observed values of three statistics (θ_w_, Tajima’s *D*, and Fu & Li’s D*) in the genome-wide analysis of RSSC phylotypes

Phylotype/replicon	Gene ID^*a*^	Gene name	Number of significant hits^*b*^	Summary statistics^*c*^			Gene description/function
				θ_w_	Tajima’s D	Fu & Li’s D*	
I/chromosome	RSc2735	*phcB*	5	0.0661**	1.6873**	1.7108**	Class I SAM-dependent methyltransferase
I/chromosome	RSc2736	*phcS*	2	0.0729**	1.6139**	1.7266**	Two-component sensor histidine kinase
I/chromosome	RSc0688	*–*	1	0.0482**	2.9588**	1.6747**	Glycosyl transferase
I/chromosome	RSc2066	*–*	4	0.0595**	3.4633**	1.7026**	Haloacid dehalogenase-like hydrolase
I/chromosome	RSc2670	*lrgB*	1	0.0154**	2.2028**	1.4372**	Effector of murein hydrolase transmembrane protein
I/chromosome	RSc2669	*lrgA*	1	0.0210**	2.4378**	1.1771*	Effector of murein hydrolase
I/megaplasmid	RSp0832	*uxuL*	1	0.0155**	1.6730**	1.4372**	Glucuronolactone/galactarolactone lactonase
I/megaplasmid	RSp0304	*ripD*	2	0.0352**	1.5729**	1.4062**	Type III effector protein, avrPphD family
I/megaplasmid	RSp0487	*ripA4*	1	0.0183**	2.5310**	1.4823**	Type III effector protein (formerly AWR4)
I/megaplasmid	RSp1212	*ripU*	2	0.1156**	1.4848**	1.4752**	Type III effector protein
I/megaplasmid	RSp0238	glgX	1	0.0296**	2.3832**	1.3369**	Probable pulA pullulanase related glycosidase protein, glycogen debranching enzyme
I/megaplasmid	RSp1530	–	1	0.0944**	1.5829**	1.6620**	Polyphenol oxidase (laccase) oxidoreductase
I/megaplasmid	RSp1100	*–*	1	0.0493**	2.1856**	1.3555**	Putative signal sensing transmembrane protein, phosphorelay sensor kinase activity
IIA/chromosome	RCFBP_11371	*paaH2*	1	0.0298**	2.0233**	1.5364**	Putative 3-hydroxybutyryl-coA dehydrogenase oxidoreductase
IIA/chromosome	RCFBP_11349	*–*	1	0.0364**	2.1164**	1.5632**	Putative high-affinity branched-chain amino acid transport system permease (liv operon)
IIA/chromosome	RCFBP_20503	*parC*	1	0.0381**	1.8461**	1.5686**	DNA topoisomerase IV, subunit A
IIA/chromosome	RCFBP_11056	*spec*	1	0.0248**	2.1501**	1.5085**	Ornithine decarboxylase
IIA/chromosome	RCFBP_10967	*ileS*	1	0.0248**	2.0846**	1.5085**	Isoleucine--tRNA ligase
IIA/chromosome	RCFBP_21311	*argC*	1	0.0232**	1.9107**	1.4970**	N-acetyl-gamma-glutamyl-phosphate reductase
IIA/chromosome	RCFBP_10305		1	0.0282**	1.9675**	1.5280**	Putative transcription regulator protein
IIA/chromosome	RCFBP_10218	*soxF*	1	0.0248**	2.0192**	1.5085**	Sulfide dehydrogenase [flavocytochrome c] flavoprotein chain precursor
IIA/chromosome	RCFBP_11858	*bioA*	1	0.0265**	1.9424**	1.5188**	Adenosylmethionine--8-amino-7-oxononanoate transaminase, PLP-dependent
IIA/chromosome	RCFBP_10092	*–*	1	0.0381**	1.9520**	1.5686**	Putative transporter, with ABC transmembrane type-1 domain
IIA/chromosome	RCFBP_10712	*phcQ*	1	0.0282**	1.9908**	1.5280**	Response regulator receiver
IIA/chromosome	RCFBP_10711	*–*	2	0.0450**	2.0770**	1.5866**	Putative methyltransferase
IIA/chromosome	RCFBP_21242	*–*	1	0.0414**	2.5492**	1.5782**	Putative isomerase, with PhzC/PhzF domain
IIA/chromosome	RCFBP_20936	*srkA*	1	0.0298**	2.0897**	1.5364**	Stress response kinase A
IIA/chromosome	RCFBP_10686	*ripW*	2	0.0911**	2.0865**	1.5603**	Type III effector protein
IIA/chromosome	RCFBP_11806	*ripG4*	2	0.0381**	2.0578**	1.5686**	Type III effector protein
IIA/chromosome	RCFBP_11870	*ripM*	1	0.0298**	2.1673**	1.5364**	Type III effector protein
IIA/chromosome	RCFBP_20594	*ripS5*	1	0.0265**	1.9424**	1.5188**	Type III effector protein
IIA/megaplasmid	RCFBP_mp10317	*cyaB*	1	0.0939**	0.9326	1.5961**	ABC transporter (cyclolysin-type)
IIA/megaplasmid	RCFBP_mp10609	*–*	1	0.0776**	1.4690*	1.1125	Putative adhesin/hemolysin
IIA/megaplasmid	RCFBP_mp30035	*cls*	1	0.2490**	1.7362**	1.6971**	Cardiolipin synthase A
IIA/megaplasmid	RCFBP_mp30119	*–*	1	0.0653**	1.2030*	1.4386**	Putative type IV fimbrial biogenesis protein pilY1 with A-like domain
IIA/megaplasmid	RCFBP_mp30438	*ripF1*	1	0.1**	1.0458	1.5313**	Type III effector protein (formerly PopF1)
IIA/megaplasmid	RCFBP_mp20003	*–*	1	0.0551**	1.6353**	1.0059	Bacteriophage-related protein of unknown function
IIB/chromosome	RSPO_c00124	*atpH*	1	0.0226**	2.1129**	1.5336**	ATP synthase, f1 sector subunit delta
IIB/chromosome	RSPO_c00113	*livK*	1	0.0211**	2.0978**	1.5182**	Leucine-specific binding precursor transmembrane protein
IIB/chromosome	RSPO_c00179	*gcl*	1	0.0240**	2.1552**	1.5475**	Tartronate-semialdehyde synthase (glyoxylate carboligase)
IIB/chromosome	RSPO_c00415 RSPO_c00416	*–*	1	0.0352**	2.0846**	1.4063**	b-ketoadipate enol-lactone hydrolase protein and 3-ketoacyl-(acyl-carrier-protein) reductase
IIB/chromosome	RSPO_c00497	*secY*	1	0.0282**	1.9898**	1.5826**	Preprotein translocase (membrane subunit)
IIB/chromosome	RSPO_c00765	*phcB*	1	0.0240**	2.1264**	1.5475**	Regulatory protein
IIB/chromosome	RSPO_c02646	*pilX*	1	0.0211**	2.0335**	1.5182**	Putative type IV pili assembly protein
IIB/chromosome	RSPO_c01209	*–*	1	0.0282**	2.7986**	1.5826**	1-deoxy-d-xylulose-5-phosphate synthase protein
IIB/chromosome	RSPO_c01332	*ripAJ*	2	0.0226**	2.2473**	1.5336**	Type III effector protein
IIB/chromosome	RSPO_c02391	*lldP*	1	0.0183**	1.9736**	1.4823**	l-lactate permease protein
IIB/chromosome	RSPO_c02306	*lepA*	1	0.0268**	2.0272**	1.5718**	GTP-binding elongation factor
IIB/chromosome	RSPO_c01998	*ripG7*	3	0.0804**	2.0276**	1.4278**	Type III effector protein (formerly GALA7)
IIB/chromosome	RSPO_c01999	*ripG6*	1	0.0268**	2.6851**	1.5718**	Type III effector protein (formerly GALA6)
IIB/chromosome	RSPO_c01798	*aidB*	1	0.0536**	2.2941**	1.3902**	Isovaleryl CoA dehydrogenase
IIB/chromosome	RSPO_c01795	*fadB*	1	0,0338**	2.0162**	1.6180**	Fused 3-hydroxybutyryl-CoA epimerase
IIB/chromosome	RSPO_c00909	*–*	1	0.0620**	3.0207**	1.5774**	Lipoprotein
IIB/chromosome	RSPO_c01066	*metG1*	1	0.0254**	2.2792**	1.5602**	Methionyl-tRNA synthetase
IIB/chromosome	RSPO_c01082	*spec*	1	0.0338**	2.5566**	1.3908**	Biodegradative ornithine decarboxylase protein
IIB/chromosome	RSPO_c03170	*–*	1	0.0354**	2.0256**	1.5602**	Chromate transport protein
IIB/chromosome	RSPO_c03029	*–*	1	0.0312**	1.9276**	1.3562**	Sensory box/GGDEF family protein
IIB/megaplasmid	RSPO_m01227	*fabI*	1	0.0466**	2.2681**	1.6703**	Enoyl-[acyl-carrier-protein] reductase (NADH)
IIB/megaplasmid	RSPO_m01150 RSPO_m01151	*pehC*	1	0.0409**	2.3366**	1.4587**	Gluconolactonase and polygalacturonase proteins
IIB/megaplasmid	RSPO_m00202	*ripH2*	9	0.0776**	2.2152**	1.7322**	Type III effector protein
IIB/megaplasmid	RSPO_m00035	*ripG3*	1	0.0676**	2.3026**	1.4768**	Type III effector protein (formerly GALA3)
IIB/megaplasmid	RSPO_m01206	*ripAO*	1	0.0494**	2.1286**	1.6788**	Type III effector protein
IIB/megaplasmid	RSPO_m01229	*ripS3*	3	0.0409**	1.9967**	1.6505**	Type III effector protein (formerly SKWP3)
IIB/megaplasmid	RSPO_m01312	*ripZ*	1	0.0747**	2.9580**	1.7286**	Type III effector protein
IIB/megaplasmid	RSPO_m01371	*ripC1*	1	0.0338**	2.5836**	1.3908**	Type III effector protein
IIB/megaplasmid	RSPO_m00869	*ripN*	1	0.0620**	2.0883**	1.5774**	Type III effector protein
IIB/megaplasmid	RSPO_m00770	*ripAR*	2	0.0380**	2.2529**	1.6388**	Type III effector protein
IIB/megaplasmid	RSPO_m01600	*ripBH*	3	0.0620**	2.7880**	1.7082**	Type III effector protein
IIB/megaplasmid	RSPO_m01541	*ripF1*	2	0.0366**	2.1843**	1.6322**	Type III effector protein (formerly PopF1)
IV/chromosome	RPSI07_1784	*–*	2	0.0312**	1.5828**	1.5828**	Putative ABC-type transporter, periplasmic component
IV/chromosome	RPSI07_2871	*tyrS*	2	0.0312**	1.5828**	1.5828**	Tyrosyl-tRNA synthetase
IV/chromosome	RPSI07_1208	*rpoD*	3	0.0768**	1.8719**	1.8719**	RNA polymerase sigma70 factor
IV/chromosome	RPSI07_1185	*galU*	2	0.048**	1.6941**	1.6941**	Glucose-1-phosphate uridylyltransferase
IV/chromosome	RPSI07_0660	*mraY*	1	0.384**	1.6419**	16419**	Phospho-N-acetylmuramoyl-pentapeptide transferase
IV/chromosome	RPSI07_0072	*ripE1_1*	2	0,1056**	1.6690**	1.6690**	Type III effector protein
IV/chromosome	RPSI07_0735	*ripW*	3	0.1056**	1.9186**	1.6690**	Type III effector protein
IV/megaplasmid	RPSI07_mp0105	*–*	1	0.1464**	1.7880**	1.7880**	Putative acetyltransferase
IV/megaplasmid	RPSI07_mp0022	*clcB*	1	0.0624**	1.6238**	1.6238**	Chloride channel clcB-like protein

^*a*^ Systematic gene identifier according to GMI1000, CFBP2957, Po82 or PSI07 strain nomenclature for phylotype I, IIA, IIB or IV respectively

^*b*^ Number of significant windows overlapping described gene

^*c*^ Observed values of statistics for each gene and significance of coalescent simulations using standard neutral model: * *p* < 0.1 and ** *p* < 0.05

In general terms, the results show that BS affects more frequently coding regions than non-coding sequences in RSSC genomes (compare Table 2 with Additional file 1: Table S3). We found 161 windows with significant values for the three summary statistics. Demography simulations reduced the number to 142 significant windows that correspond to 78 known genes (Table 1) and 22 intergenic regions or genes with unknown identity or function (Additional file 1: Table S3). This may indicate that 19 windows are probably false positives. The percentage of genes with known function detected under BS is low (1.7%, 78 genes out of 4594 total genes which is the median number of protein-coding genes in RSSC according to the Genome Database, https://www.ncbi.nlm.nih.gov/genome/microbes/). The candidate genes under BS are described below according to phylotype and replicon.

*Phylotype I.* We detected 440 windows for the chromosome of this phylogenetic group at the top 5% of the distribution, however only 21 showed concurrent high values in all three summary statistics and 14 were recorded as highly significant after the simulation process.

**Fig. 2:**
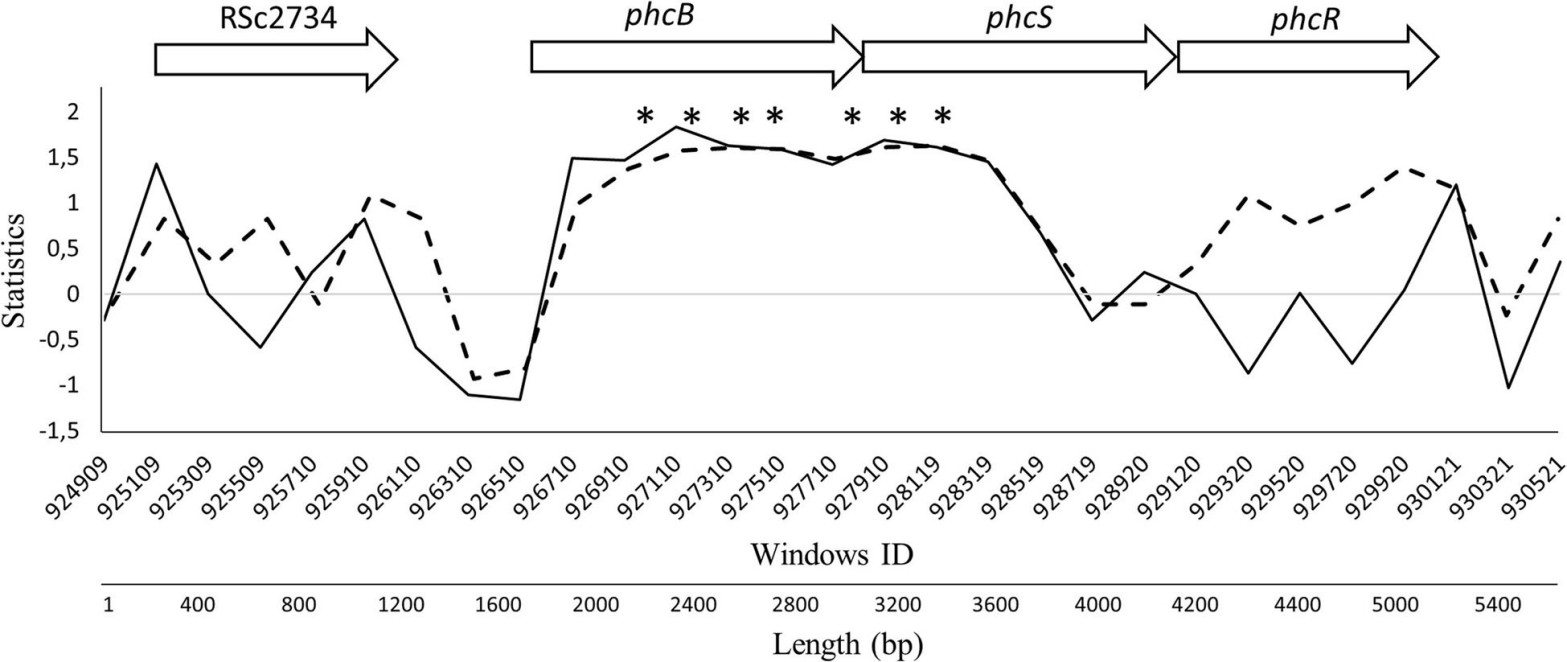
Analysis of genomic region corresponding to the *phcBSR* operon in strain GMI1000 showing sliding window analyses for two statistics: Tajima’s D (solid line) and Fu & Li’s D* (dotted line). All three genes of the operon comprise about 3.9 Kb of the genome; however, the gene (RSc2734) physically preceding this operon is also shown for comparison purposes. Windows are 200 bp and asterisks indicate the windows with extreme values of respective statistics. Arrows represent gene arrangement in the genome of strain GMI1000

We found five and two extreme values of Tajima’s D, θ_w_ and Fu & Li’s D statistics on genes *phcB* and *phcS*, respectively (Fig. 2, Table 2). These two genes are arranged in an operon together with a third gene named *phcR*. The gene *phcB* encodes a SAM-dependent methyltransferase that synthesizes methyl 3-hydroxypalmitate (3-OH PAME) or methyl 3-hydroxymyristate (3-OH MAME), a quorum-sensing signal that accumulates in the extracellular space when the bacteria are multiplying rapidly in a restricted space [29]. Quorum-sensing is a key process regulating and synchronizing the expression of specific genes involved in biofilm formation, pathogenicity, and production of secondary metabolites like siderophores, exoproteases, and exotoxins [30]. Genes *phcS* (histidine kinase) and *phcR* (response regulator) code for elements of a two-component regulatory system that responds to threshold concentrations of 3-OH PAME or 3-OH MAME by elevating the level of functional PhcA, the fourth component of the system [31, 32]. PhcA is the global virulence regulator in RSSC that coordinates the expression of several virulence-related genes including those responsible for biosynthesis of the major EPS, cell wall degrading enzymes, T3SS effectors, and others representing a total of 383 genes [33, 34].

Another gene showing multiple peaks in statistics values is RSc2066 that codes for a haloacid dehalogenase-like hydrolase (Table 2). In this case, four consecutive high values of the statistics suggest that this gene is likely under BS. This enzyme has a hydrolase activity that cleaves different bonds (i.e. C-O, C-N, C-C), however its exact role at the cellular level is unknown.

In this phylotype we also found genes related to basic metabolism like a glycosyl transferase and an operon consisting of two genes, *lrgAB*, that modulates murein hydrolase activity which is linked to biofilm dispersal and cell lysis [35]. These *lrgAB* genes intervene indirectly in pathogenesis since an essential step in this process is the formation and dispersal of biofilms in RSSC [30].

For the megaplasmid we found 304 out of 6162 windows with highest Tajima’s D, θ_w_, and Fu & Li’s D* values. After simulation for relevant demographic models, only nine windows generated significant values.

Some interesting genes associated to virulence were observed in this replicon (Table 2). We identified three different T3SS effector genes as targets for BS: *ripD* of the avrPphD family; *ripA4* and *ripU*. Interestingly, both *ripD* and *ripU* show two significant hits (two windows with significant values) along their coding sequences. *RipU* is part of the core-effectome within the RSSC as well as *ripA4* that is common in effector collections and plays an important role in the interaction between *R. solanacearum* and the pepper plant [36]. Another gene, *uxuL* (RSp0832) codes for the main glucuronolactone/galactarolactone lactonase in the genome of the GMI1000 strain. UxuL is organized within an operon with three other genes: *garD* encodes a D-galactarate dehydratase, RSc0831 a putative NAD-dependent epimerase/dehydratase and *pehC* a polygalacturonase. PehC is an enzyme related to virulence since it cleaves oligomers of galacturonate, however its exact role is unknown. It was hypothesized that PehC acts by degrading plant oligogalacturonate signal molecules that elicit production of reactive oxygen species (ROS) as a defense response. This degradation would reduce tomato antimicrobial responses and increase bacterial virulence [37]. This operon is regulated by GulR, a transcription factor of the LysR family involved in glucuronate utilization and metabolism. Downstream of this operon is located *exuT*, the galacturonate transporter gene.

Conversely, genes that are not directly related to virulence but to primary metabolism were also identified in megaplasmid aligned sequences: A probable PulA pullulanase related glycosidase protein that might work like a glycogen debranching enzyme; a polyphenol oxidase (laccase) and a putative signal sensing transmembrane protein with phosphorelay sensor kinase activity. Lastly, a significant window matched with an intergenic region surrounded by a hybrid sensor histidine kinase/response regulator and upstream of an integrase related to phage or transposon insertion (Additional file 1: Table S3).

*Phylotype IIA.* At the chromosome level we selected 444 windows that showed 5% highest scores in each summary statistic. From these, 21 windows showed highest values for all three statistics and also significant values on simulations with the respective demographic models.

The first genes that appear in the list are those involved in essential cell functions. There are various enzymatic functions (i.e. a 3-hydroxybutyryl-coA dehydrogenase oxidoreductase, an isoleucine--tRNA ligase, a transcription regulator and others, Table 2) and diverse transporters (a permease from the *liv* operon, a binding-protein-dependent transporter). Among this group, a gene that attracted our attention encodes an ornithine decarboxylase, which is a homolog of the *Escherichia coli* SpeC decarboxylase in GMI1000 strain [38]. This gene and other related genes (arginine and lysine decarboxylases) are directly involved in amino acid metabolism but indirectly in pathogenesis. Studies on other bacterial species indicate that these genes are implicated in stress response against the low pH in the medium [39, 40] and against oxidative stress and chemical quenching induced by the host [41]. Metabolic products of amino acid decarboxylases also intervene in cell adhesion to host tissues [42].

Among the genes related to virulence and survival, we found two contiguous genes, *phcQ* and another one downstream from it showing elevated values of selection statistics. PhcQ is a response regulator receiver, from the CheY family and part of the *phcBSRQ* operon that regulates PhcA, the master regulator that positively and negatively regulates many genes responsible for pathogenicity in RSSC [43]. The gene contiguous to *phcQ* encodes a methyltransferase, however it is not known if PhcQ participates in quorum sensing as does the main methyltransferase, PhcB. Two additional genes were associated to BS signatures: *srkA* and RCFBP_21242. The *srkA* gene encodes a stress response kinase A, which probably counteracts the accumulation of ROS produced by the host and protects the bacterial cell from antimicrobial and environmental stressors in a similar way to the YihE protein kinase of *Escherichia coli* [44]. RCFBP_21242 encodes a putative isomerase with a phenazine biosynthesis (PhzC/PhzF) domain. Phenazines constitute a large group of nitrogen-containing heterocyclic compounds produced by bacteria and show an ability to handle ROS, contribute to biofilm formation, cell adhesion and enhance bacterial survival, among other activities [45].

Results from the analysis of the T3SS effector repertoire of phylotype IIA-chromosome showed a number of genes with a BS signature: *ripM*, *ripW*, *ripG4* (formerly GALA4) and *ripS5* (formerly SKWP5). *RipG4* and *ripW* were associated to two significant windows each suggesting these genes are clearly under BS. Since we have used the CFBP2957 strain as a reference for gene identification, we find that this strain has an insertion of a transposon encoding a transposase (RCFBP_20595) in the *ripS5* gene, therefore this appears to be a pseudogene copy of this effector. Most of the phylotype IIA strains show a disruption in the *ripS5* gene due to transposon insertions, however there are some strains harboring the complete gene (i.e. the RS_489 strain, [46]).

At the megaplasmid level, phylotype IIA showed six genes with significant signatures of BS after filtering with coalescent simulations: one related to basic metabolism (*cyaB*, an ABC transporter) and four pertaining to pathogenicity: a putative adhesin/hemolysin that plays a significant role in cell adhesion; a cardiolipin synthase A, from the phospholipase D family, involved in membrane biosynthesis and toxin production and resistance [47]; a putative Type IV fimbrial component, encoded by the *pilY*1 gene participating in Type IV pili biosynthesis. Type IV pili are essential for adhesion and pathogenesis [48]. Finally, a T3SS effector named *ripF1* (formerly PopF1) that is very well characterized [49] and a bacteriophage-related protein with unknown function.

*Phylotype IIB.* Three hundred fifty two windows corresponding to the top 5% of the distribution were analyzed for genes located on the chromosome. As explained in Methods, only 33 windows showed highest values of the three summary statistics concurrently, but 23 windows showed significant values after coalescent simulations.

The most abundant group of genes identified in this chromosome are those involved in primary metabolism with an ample diversity that varies from genes encoding metabolic enzymes (synthases, epimerases, etc.) to a number of permeases and other transporter related genes (Table 2). Again, an amino acid decarboxylase was found within this group.

Various genes are linked to virulence. Again, a key component of pili biogenesis (Type IV pili assembly protein PilX) and the gene responsible for the production of 3-OH PAME or 3-OH MAME, which mediates quorum sensing, *phcB,* were identified. Among genes encoding T3SS effectors, three were most notable (*ripAJ*, *ripG6* and *ripG7*) and multiple windows enriched two of them (two and three hits for *ripAJ* and *ripG7*, respectively, Table 2). Interestingly, a conserved protein (RSPO_c02827) showed also two significant hits along its sequence but its function is unknown (Additional file 1: Table S3).

We identified 26 windows with significant values distributed across the megaplasmid after the simulation process. Since many virulence-related genes reside in the megaplasmid, it was not surprising to have identified many of them. Ten different T3SS effector genes were found (Table 2) and some were noted by redundant windows as is the case of genes *ripH2* (9 hits), *ripS3* (3 hits), *ripBH* (3 hits), *ripAR* (2 hits) and *ripF1* (2 hits). On the other hand, only few genes involved in basic metabolism were identified: an enoyl reductase (NADH dependent) and two contiguous genes, polygalacturonase and gluconolactonase, that overlap within a single window (N-terminus of the first and C-terminus of the second enzyme).

*Phylotype 4.* The chromosome showed 463 windows in the top 5% of the distribution for each summary statistic, and after selection for the matching values in the three statistics and the simulation, only 15 were retained as highly significant for further analyses.

We found interesting genes in the chromosome such as one encoding the RNA polymerase sigma 70 factor which gathered three consecutive windows. Other genes that received multiple hits include a tyrosyl-tRNA synthetase, a glucose-1-phosphate uridylyltransferase and a putative ABC-type transporter. On the other hand, a phospho-N-acetylmuramoyl-pentapeptide transferase was detected by one window. In the gene group related to virulence, we found two T3SS effector genes with multiple windows: *ripE1* from the AvrPphE family and *ripW* (formerly PopW), a hairpin with a pectate lyase domain.

At the megaplasmid level, we found only two metabolically essential genes with significant values: a putative acetyltransferase and a chloride channel clcB-like protein.

Finally, analysis of nucleotide substitutions in the third position of the codon identified positively selected sites in the genes under BS. Most of the genes showed sites under positive diversifying selection (64 out of 78 genes) with a number of sites that varies from 1 to 116 and an average of 7.7 sites/gene (see Additional file 1: Table S5).

## Discussion

In this work, we report the systematic exploration of the genomes belonging to the main RSSC phylotypes with the intention of finding signatures of BS. To our knowledge this is the first time that a bacterial plant pathogen is analyzed for this type of selection at the genomic level. The analysis was performed on the main replicons of RSSC (chromosome and megaplasmid), but not on small plasmids, phages or mobile genetic elements. We scanned genome sequences using a sliding window approach and subsequently applied widely used summary statistical tests aimed at detecting the excess of polymorphisms on 200 bp-window sequences: Watterson estimator theta, Tajima’s D, and Fu & Li’s D*. We chose to use these tests rather than other strategies (i.e. model based methods) because of their simplicity, wide range of BS forms detected and broad access to diverse software tools. This strategy together with exhaustive coalescent simulations to correct confounding effects of demography was an effective approach to reach our objective to detect genes and genomic regions under BS in RSSC. Tajima’s D is useful for detecting intermediate and ancient signatures of BS. In contrast Fu & Li’s D* and θ_w_ help to identify relatively recent instances of this type of selection. Our approach may be overly conservative, and hence we might have missed some genuine occurrences of BS. On the other hand, it may have conferred more certainty to the positive hits found on RSCC genomes. Indeed, we detected dozens of gene candidates in RSSC genomes in agreement with Fijarczyk and Babik [50] who recognized this is common in pathogens’ genomes.

The results add new insights to understand the RSSC-plant host interactions. We found 78 and 11 genes with known and unknown function respectively (totaling 89 genes or 1.9% of the total genes in RSSC). This result is consistent with other analyses in eukaryotic systems like humans [8, 51] or plants [9] that stress the rarity of finding BS signatures on sequence genomes. In the case of bacteria, an article devoted to finding BS signatures in the *S. aureus* genome indicates that 5.7% of the examined genes show BS [11]. We have found many bacterial genes that show unambiguously features of being under BS. The *phcBRS* operon scored 7 significant windows in phylotype I and one in phylotype IIA as well as one in phylotype IIB, indicating this genomic region is under strong BS. Remarkably, Guidot and collaborators [52] also found that one component of this system, *phcS*, was subject to strong selection from the plant host given the evidence that this gene was targeted by mutations in an *in planta* experimental evolution system. In this connection, an equivalent to *phcBRS* system but simpler network in *S. aureus*, the *agr* locus, is also a two-component signal transduction system (membrane-bound histidine kinase sensor, AgrC and transcriptional regulator, AgrA), with a signal molecule (an auto-inducing peptide, AgrD) and a protein responsible for the maturation and export of the signal molecule (AgrB). Again, the key component in this system is the master transcriptional regulator AgrA that binds two divergent promoters, P1 and P2 [53]. Although this system does not show homology at the sequence level with the *phcBRS* system in RSSC, it is functionally analogous since it leads to up and down-regulation of over 70 genes, 23 of which are known to be directly related to virulence [54]. Interestingly, the *agr* locus has the strongest known signatures of BS in bacteria to date due to the high number of common polymorphisms. For this reason, the *agr* locus has been proposed as the positive control of BS for further studies in bacteria [11].

We have also found a set of genes showing strong BS signatures related to adhesion, motility and biofilm formation. Genes encoding Type IV fimbrial biogenesis proteins (*pilY1* in phylotype IIA/megaplasmid and *pilX* in phylotype IIB/chromosome) are essential for the assembly and function of Type IV pili, filamentous structures that mediate bacterial adhesion to surfaces including host cells. This adhesion is tightly linked to the bacterial pathogens’ ability to promote the formation of microcolonies and biofilms as well as to their twitching motility and virulence [55, 56]. The *lrgA* and *lrgB* genes (phylotype I/chromosome) are required for biofilm dispersal that is accompanied by cell lysis and death [35]. Biofilm formation and disruption is a critical step in the process of infection and pathogenesis for RSSC strains*.* Diverse types of molecules mediate the release of the cells from biofilms, including degrading enzymes (among them, murein hydrolases), nucleases and others [57, 58]. Additionally, we identified one gene under BS that seems to be directly related to the biosynthesis of phenazines in phylotype IIA. Phenazines constitute a large group of nitrogen-containing heterocyclic compounds produced by a wide range of bacteria, with diverse physiological functions. Among these, they influence swarming motility and biofilm architecture through a not fully understood mechanism [59].

T3SS effectors are key virulence factors at the forefront of the arsenal that RSSC strains harbor to infect plants and achieve full pathogenicity including the metabolic adaptation to parasitic life in the plant [60]. T3SS effectors are delivered to plant cells through a proteinaceous needle-like structure, and once inside, they manipulate plant cell metabolism to suppress or evade defense responses and promote bacterial multiplication [61]. *R. solanacearum* strains possess a large repertoire, with 118 effectors identified among RSSC sequenced [46, 62]. We found 21 different T3SS effector genes with moderate to very strong BS signatures in all phylotypes studied here (Table 2). The percentage of T3SS effectors under BS relative to the total number of T3SS effectors is: Phylotype I: 5%, Phylotype IIA: 27.7%, Phylotype IIB: 27% and Phylotype IV: 7%. Some T3SS effector genes belong to very well-known families of effectors like the GALA (*ripG4*, in phylotype IIA, chromosome; *ripG6* and *ripG7* in phylotype IIB, chromosome; *rip*G3, in phylotype IIB, megaplasmid), SKWP (*rip*S5 in phylotype IIA, chromosome; *rip*S3, in phylotype IIB, megaplasmid), HLK (*rip*H2 in phylotype IIB, megaplasmid) and PopF type III translocators (*rip*F1). Interestingly, there are a number of cases in which sequences of two or more windows correspond to the same T3SS effector gene from the same phylotype (*ripD* in phylotype I, megaplasmid; *ripU* in phylotype I, megaplasmid; *rip*W and *rip*G4 in phylotype IIA, chromosome; *ripAJ* and *rip*G7 in phylotype IIB, chromosome; *ripH2*, *ripS3*, *ripAR*, *ripBH* and *ripF1* in phylotype IIB, megaplasmid; *ripE1_1* and *ripW* in phylotype IV, chromosome) or from different phylotypes (*rip*W, in phylotype IIA, chromosome and phylotype IV, chromosome). This co-localization of windows on same gene provides strong evidence that these genes are under BS.

Although genes dedicated to tasks of basal metabolism may seem less relevant for pathogenesis, they also play an important role in the interaction with the plant host and virulence. Peyraud and collaborators [63] developed a model system to study robustness and metabolic responses to internal and environmental perturbations in *R. solanacearum*. One of their findings highlights the active participation of primary metabolism in sustaining virulence by activating functionally redundant reactions which may require redundant alleles to satisfy cellular demands including virulence. The expression of virulence factors (such as the exopolysaccharide) is controlled by the virulence regulatory network (VRN) that operates with overlapping genes or operons involved in amino acid synthesis [63]. While we did not particularly seek redundant or duplicate alleles in this work, we found a number of genes of primary metabolism that perform similar functions at the cellular level. For example, in the set of genes showing BS signatures there are two glucuronolactonases (carbohydrate metabolism), two aminoacyl-tRNA synthetases and two aminoacyl-decarboxylases (amino acid metabolism). These genes participate in primary metabolism and probably are indirectly playing an essential role in virulence. Another group of genes that we should not neglect are those involved in defense and reduction of toxicity by metabolites produced by the plant host defense mechanisms. In the list of candidate genes under BS, we can count a stress response kinase A (*srkA*) and a number of membrane transporters (ABC transporters and other permeases, see Table 2). Genes participating on defense pathways were also enriched in *S. aureus* genome analysis for BS signatures [11].

Interestingly, we found evidence that some individual sites in the RSSC genes under BS are subject to positive diversifying selection. We used the MEME analysis, which is recommended for the identification of positive diversifying selection in gene sites because it is sensitive to cases of transient or episodic selection [64]. This result confirms that sites in the genes showing BS signature are, indeed, under positive selection, either balancing or diversifying.

In summary, more than a half of the identified genes with BS are devoted to primary metabolism and other activities such as stress response and the rest of the genes (48.7%) correspond to virulence-related genes including T3SS effectors.

## Conclusions

In this study, we present an analysis of BS operating in a major plant pathogen. This analysis is particularly relevant to understand the dynamics of plant-microbe interactions. Pathogens create and maintain a high variation of polymorphisms (detected as BS) in virulent and avirulent genes focused on contributing to the pathogenicity process [5]. Interestingly, in RSSC genomes, we found high variation in T3SS effector genes and other virulence-related genes as measured by Tajima’s D and other complementary summary statistics (Table 2), which may be under significant selection pressure by the plant host. Considering that RSSC has the ability to infect a large number of different plant species [21], it is not rare to find this high variation in the virulence factors. Some effectors (the so-called avirulence proteins) are recognized by proteins encoded by the plant R genes, however escape from host recognition is possible through fixing mutations on genes coding for effectors or other virulence proteins that increase variation. In order to evade plant detection and defense response, RSSC may tend to favor the maintenance of various allele alternatives (observed in the form of BS), which at the same time increases pathogen fitness. In a more applied sense, the identification of genes under BS, as illustrated in this work, opens the possibility to develop strategies towards establishing long term resistance or tolerance to pathogens in plants. These genes are potential targets for plant immunity, hence potential candidates to engineer broad disease resistance in agriculturally relevant plants.

## Methods

### Sequence data and alignment

Fifty-seven full-genome sequences of three RSSC phylotypes were downloaded from NCBI’s FTP server (https://www.ncbi.nlm.nih.gov/genome/microbes/) in February and April 2018. We selected 20 genomes for phylotype I (CQPS-1, FJAT-1458, FJAT-91, FQY_4, GMI1000, KACC10709, OE1–1, PSS1308, PSS190, PSS4, RD13–01, RD15, Rs-09-161, Rs-10-244, Rs-T02, SD54, SEPPX05, TO10, UW757, YC45) and phylotype IIB (23-10BR, CFBP1416, CFBP3858, CFBP6783, CFBP7014, CIP417, GEO_304, GEO_96, IBSBF1503, IPO1609, Po82, RS 488, RS2, UW163, UW179, UW24, UW365, UW491, UW551, UY031). For phylotypes IIA and IV we used the largest number of genomes available in the database (12 genome sequences: B50, BBAC-C1, CFBP2957, CIP120, Grenada 9–1, IBSBF1900, K60, P597, RS 489, UW181, UW25, UW700; and 5 genome sequences: A2-HR MARDI, KACC 10722, PSI07, R229, R24, respectively; see Additional file for genome sequence identifiers). Unfortunately, there were not enough genome sequences for phylotype III at the time we retrieved sequences to perform analyses, therefore we did not include this phylotype in the analysis. All analyses were performed on the main (chromosome) and the secondary (megaplasmid) replicons of the RSSC.

We aligned the genome sequences using progressiveMauve aligner v2.4.0 [65] with default settings. For phylotype IV sequences, we increased the gap penalty (gap open score − 600) to avoid opening unnecessarily large gaps, however we allowed small gaps (3–10 bp). For all analyses, we used only Locally Collinear Blocks (LCBs, ≥5000 bp in length) sequences to assure we worked with homologous sites that show maximal collinearity in order to avoid problems of internal genome rearrangements and gene gain and loss. We used stripSubsetLCBs script distributed with Mauve to extract LCBs longer than 1000 bp that were shared by RSSC genomes. This script generates an xmfa file that should be converted to a fasta file to facilitate the ensuing analyses. For this purpose, we used a Perl script (xmfa2fasta).

### Statistical analyses

We applied summary statistics to detect BS. The summary statistics were used to measure an excess of polymorphisms linked to the genomic regions under this type of selection. We adopted three different summary statistics: Watterson’s estimate of theta (θ_w_), Tajima’s D, and Fu & Li’s D* [66, 67]. Tajima’s D test takes into account the average pairwise nucleotide diversity between sequences and the number of segregating sites expected under neutrality for a population at mutation-drift equilibrium [68]. Tajima’s D is useful to detect departures from neutrality when considering an excess of rare alleles indicating positive selection/selective sweep, or the opposite, excess of common alleles that leads to assume BS has operated in the population. In our case, Tajima’s D helps to find polymorphisms at intermediate frequency. Watterson’s theta measures the population mutation rate, which is understood as the product of the effective population size, and the neutral mutation rate from the observed nucleotide diversity of a population [27]. In this case, θ_w_ is an indicator of high level of polymorphisms. Fu & Li’s D* statistics considers the number of derived singleton mutations and the total number of derived nucleotide variants without an outgroup [69]. We used a combination of these three test statistics to detect excess of common polymorphisms along the allele frequency spectrum relative to expectations under neutral equilibrium. The use of three indicators may seem overly conservative, but it helps to reduce false positives and to detect genes or genome regions that are robust candidates for operating under BS. Neutrality tests were calculated with VariScan 2.0.3 [70] using total number of segregating sites and excluding sites containing gaps or ambiguous nucleotides.

We performed a genome-wide scan to find genes or genome regions under BS using a sliding window approach. Thomas and colleagues [11] tested windows of two sizes, 100 bp and 200 bp for *S. aureus* genome analysis coming to the conclusion that 200 bp windows is the optimal and 100 bp windows is the second best alternative for a genome scan. The type strain of *S. aureus* subsp. *aureus* DSM 20231^T^ has a genome of 2,9 Mb [71] which is slightly smaller than the RSSC chromosome (3.7 Mb for reference strain GMI1000, [23]). Moreover, the average length of protein-coding genes is similar for both bacterial species (946 bp for chromosome, 1077 bp for megaplasmid for RSSC and about 1009 bp for *S. aureus*; [23, 71]). Therefore, a 200 bp window seems to be an adequate window size for RSSC. All three statistics were calculated for consecutive, non-overlapping 200 bp windows, and only those windows with the highest 5% values coinciding in the three statistics were chosen as possible candidates for further analyses. Windows without single nucleotide polymorphisms (SNPs) among aligned genomes were excluded from analysis because the statistics are calculated based on polymorphisms.

Per site mutation (θ) and recombination (ρ) rates are parameters useful for understanding the recent history of RSSC populations, however they also help to test demographic models to discover which one best fits the observed data for each population (see below). These parameters were estimated using a penalized approximate likelihood coupled to a Bayesian reversible-jump Markov chain Monte Carlo sampling scheme. For this, we set up the starting ρ value to 30, penalized each block with a value of 10 and used the gene conversion model. We run 10^6^ chains to obtain ρ and θ values using the program INTERVAL [72] implemented in the RDP4 package [73]. Because RDP was not designed to handle long genomic sequences, we estimated values of ρ and θ by averaging the obtained values from sets of 50,000 bp each along the length of nucleotide sequence alignments.

The summary statistics (θ_w_, Tajima’s *D*, and Fu & Li’s D*) must be carefully analyzed because different demography scenarios could give similar signals as BS when applied to real population data. For example, different population structures like a contraction or a selective bottleneck could generate confounding indications mimicking BS. To correct potentially confounding effects of demography we need to select adequate null demographic models and test them with real data. For this purpose, we adopted a simulation-based approach to generate genetic statistics under three main demographic scenarios: standard neutral model (SNM), a recent population contraction model (PCM), and a recent bottleneck model (BNM). The SNM assumes a constant-sized population, thus Tajima’s D is expected to be zero [68]. Under PCM and BNM assumptions, Tajima’s D is positive or shows higher values than with SNM, which indicates the abundance of prevalent lineages before a contraction or a bottleneck effect. Simulations were performed under the coalescent simulation framework by employing the algorithm described in [74] to infer the coalescent tree with recombination. The PCM assumes that the population has undergone a size reduction at a given time that we fixed at 0.005 coalescent time units before the present, according to Thomas et al. [11]. Coalescent time units are measured in 4*N*_*e*_ generations where *N*_*e*_ corresponds to the current effective population size [74]. For BNM simulations, the model assumes that the population suffered two demographic events, a contraction and then a population growth. In this case, we calibrated time (*T*_*c*_ and *T*_*r*_ time of contraction and time of recovery, respectively) for first and second events as 0.005 coalescent time units before the present until a relevant demographic event [11]. The reduction of population size (*N*_*e*_) relative to constant growth was set to 5, for PCM and for the first and second demographic events of BNM. The fivefold reduction of the original population size is based on the *N*_*e*_ decrease reported in experimental studies performed on different bacterial species [75–78]. Finally, ρ and θ values calculated previously were used to complete the information required to run the simulations. For each window, we computed 10,000 coalescent simulations using DNASP v. 6.11.01 for the three summary statistics under the relevant demographic model [79]. A *p*-value was estimated for each window to validate statistically the potential differences between simulated and observed data. Windows with extreme (i.e. significant) *p*-values (at the right tail, *p*_Sim *< Obs*_ < 0.1 or *p*_Sim *< Obs*_ < 0.05) for the three statistics and the three demographic models were recorded as highly significant and accepted as candidates under BS. However, windows with significance for only two statistics (Tajima’s *D* and Fu & Li’s D* or Tajima’s *D* and θ_w_, see Table 2 and Additional file 1: Table S3) were also accepted as secondarily significant. We show a graphic comparison of observed and simulated data for the three statistics for Phylotype I/chromosome as an example (see Additional file 1: Figure S1).

We calculated the nucleotide diversity (π) and the ratio (ω) of non-synonymous (*Ka*) to synonymous (*Ks*) substitutions rate of the genes under BS. For π, we employed the DNASP v. 6.11.01 software [79] and for ω we used the web server https://www.datamonkey.org/meme, which implements the method called ‘mixed effects model of evolution’ (MEME) that is useful for identifying positive selection at the level of individual sites in genes [64].

### Gene identification and function

Sequences of windows with significant values were used to identify genes that overlap in them. For this, Blastn searches were performed using standard settings [80]. We used four RSSC reference strains for sequence comparison and gene identifier assignation: GMI1000 for phylotype I; CFBP2957 for phylotype IIA; Po82 for phylotype IIB; and PSI07 for phylotype IV. Uniprot [81] and Pfam [82] databases including their tools were used to retrieve information on the features and function of proteins. The respective gene ontology (GO) term was applied to each identified protein using QuickGO (https://www.ebi.ac.uk/QuickGO/). The KEGG database was used for further understanding putative gene functions, utilities of the bacterial systems and to define orthologs for RSSC genes under BS [83]. Identification of T3SS effector proteins was achieved using the web interface named “Ralstonia T3E” (https://iant.toulouse.inra.fr/T3E) with the curated effector repertoire database [46].

## Additional file

Additional file 1: **Table S1.** Summary statistics of nucleotide site frequency spectrum for each phylotype and replicon of RSSC. **Table S2.** Two-dimensional plot of three summary statistics calculated using sliding window data. **Table S3.** List of genes with unknown function and intergenic regions showing highest observed values of three statistics (θ_w_, Tajima’s *D*, and Fu & Li’s D*) in the genome-wide analysis of RSSC phylotypes. **Table S4.** Sequence identifiers of genomic data used in this study. **Table S5.** Estimation of nucleotide diversity and nonsynonymous to synonymous substitution rate ratio of RSSC genes under BS. **Figure S1.** Observed versus simulated values of summary statistics for Phylotype I/chromosome. SNM, PCM and BNM indicate simulations under three different demographic scenarios: standard neutral model, population contraction model and bottleneck model, respectively. Asterisk indicates significant *p*-values (0.05) for the respective comparisons. (DOCX 82 kb)

## Data Availability

All data generated or analyzed during this study are included in this manuscript and its Additional file. Genome sequence identifiers are supplied (Additional file 1: Table S4) that allow retrieval from public databases.

## Abbreviations

3-OH MAME: methyl 3-hydroxymyristate
3-OH PAME: methyl 3-hydroxypalmitate
BNM: Bottleneck model
BS: Balancing selection
EPS: Extracellular polysaccharide
GO: Gene ontology
LCBs: Locally Collinear Blocks
*N*_*e*_: population size
PCM: Population contraction model
ROS: Reactive oxygen species
RSSC: *Ralstonia solanacearum* species complex
SNM: Standard neutral model
SNPs: Single Nucleotide Polymorphisms
T3SS: Type III Secretion System
*T*_*c*_: Time of contraction
*T*_*r*_: Time of recovery
VRN: Virulence regulatory network
