# Supplementary material for: A genome-wide scan for genes under balancing selection in the plant pathogen Ralstonia solanacearum
Source: BMC Evol Biol. 2019 Jun 17;19:123. doi: 10.1186/s12862-019-1456-6 (PMC6580516; doi:10.1186/s12862-019-1456-6)
Supplement: Supplementary file 1 — Table S1. Summary statistics of nucleotide site frequency spectrum for each phylotype and replicon of RSSC. Table S2. Two-dimensional plot of three summary statistics calculated using sliding window data. Table S3. List of genes with unknown function and intergenic regions showing highest observed values of three statistics (θw, Tajima’s D, and Fu & Li’s D*) in the genome-wide analysis of RSSC phylotypes. Table S4. Sequence identifiers of genomic data used in this study. Table S5. Estimation of nucleotide diversity and nonsynonymous to synonymous substitution rate ratio of RSSC genes under BS. Figure S1. Observed versus simulated values of summary statistics for Phylotype I/chromosome. SNM, PCM and BNM indicate simulations under three different demographic scenarios: standard neutral model, population contraction model and bottleneck model, respectively. Asterisk indicates significant p-values (0.05) for the respective comparisons. (DOCX 82 kb) [file 12862_2019_1456_MOESM1_ESM.docx]

**Supplementary material**

A genome-wide scan for genes under balancing selection in the plant pathogen *Ralstonia solanacearum*

José A. Castillo^1*^ & Spiros N. Agathos^1^

^1^School of Biological Sciences and Engineering, Yachay Tech University, Hacienda San Jose s/n and Proyecto Yachay, Urcuquí, Ecuador.

*Corresponding Author
jcastillo@yachaytech.edu.ec

**Supplementary Table 1.** Summary statistics of nucleotide site frequency spectrum for each phylotype and replicon of RSSC.

|  | **Tajima's D** | | | **θ_W_** | | | **Fu & Li's D*** | | |
| --- | --- | --- | --- | --- | --- | --- | --- | --- | --- |
| **Phylotype/**  **replicon** | **Highest value** | **Lowest value** | **Media value** | **Highest value** | **Lowest value** | **Media value** | **Highest value** | **Lowest value** | **Media value** |
| I/chromosome | 3.4633 | -2.5060 | -0.3099 | 0.0789 | 0.0014 | 0.0056 | 1.7266 | -3.8352 | -0.4272 |
| I/megaplasmid | 2.5310 | -2.5673 | -0.3166 | 0.1395 | 0.0014 | 0.0070 | 1.6620 | -4.0969 | -0.4285 |
| IIA/chromosome | 2.7470 | -2.2010 | 0.8652 | 0.1027 | 0.0017 | 0.0103 | 1.6397 | -2.8045 | 0.7222 |
| IIA/megaplasmid | 2.4308 | -1.6949 | -0.5583 | 0.2490 | 0.0020 | 0.0162 | 1.6971 | -1.7987 | -0.6115 |
| IIB/chromosome | 3.0226 | -2.3402 | 0.6499 | 0.0888 | 0.0014 | 0.0082 | 1.6966 | -3.8403 | 0.3405 |
| IIB/megaplasmid | 3.0087 | -2.3765 | 0.6743 | 0.1409 | 0.0014 | 0.0117 | 1.7475 | -4.0058 | 0.3506 |
| IV/chromosome | 2.2454 | -1.2638 | -0.3198 | 0.1848 | 0.0024 | 0.0110 | 1.8719 | -1.2638 | -0.3377 |
| IV/megaplasmid | 2.6745 | -1.2626 | -0.3512 | 0.2616 | 0.0024 | 0.0138 | 1.7880 | -1.2626 | -0.3785 |

**Supplementary Table 2.** Correlation between the three summary statistics calculated using sliding window data.

|  | **R_S_** | | |
| --- | --- | --- | --- |
| **Phylotype/**  **replicon** | **θ_W_-Tajima's D** | **Tajima's D-Fu & Li's D*** | **θ_W_-Fu & Li's D*** |
| I/chromosome | -0.013ns | 0.809** | -0.028** |
| I/megaplasmid | 0.012ns | 0.792** | -0.033* |
| IIA/chromosome | 0.133** | 0.738** | 0.178** |
| IIA/megaplasmid | -0.068** | 0.964** | -0.099** |
| IIB/chromosome | 0.103** | 0.748** | 0.108** |
| IIB/megaplasmid | -0.158** | 0.764** | 0.127** |
| IV/chromosome | -0.054** | 0.982** | -0.07** |
| IV/megaplasmid | -0.019ns | 0.976** | -0.041* |

* and ** indicate significant differences at *p*<0.05 and *p*<0.01, respectively; ns indicate

non-significant differences, as determined using Spearman's correlation coefficient (R_s_)

**Supplementary Table 3.** List of genes with unknown function and intergenic regions showing highest observed values of three statistics (θ_w_, Tajima’s *D*, and Fu & Li’s D*) in the genome-wide analysis of RSSC phylotypes.

| **Phylotype/**  **replicon** | **Gene ID*^a^*** | **Number of significant hits*^b^*** | **Summary statistics*^c^*** | | | **Gene description** |
| --- | --- | --- | --- | --- | --- | --- |
|  |  |  | **θ_w_** | **Tajima’s D** | **Fu & Li’s D*** |  |
| I/megaplasmid | RSp1676 - RSp1677 | 1 | 0.0226 | 2.3992** | 1.5336** | Intergenic region |
| IIA/chromosome | RCFBP_11263 - RCFBP_11264 | 1 | 0.0613** | 1.9057** | 1.6158** | Intergenic region |
| IIA/chromosome | RCFBP_10695 | 1 | 0.0248** | 2.0323** | 1.5085** | Conserved hypothetical protein |
| IIA/chromosome | RCFBP_10153 | 1 | 0.0248** | 1.9013** | 1.5085 | Conserved hypothetical protein |
| IIA/chromosome | RCFBP_21040 - RCFBP_21042 | 1 | 0.0331** | 2.0892** | 1.5509** | Intergenic region |
| IIA/chromosome | RCFBP_20766 | 1 | 0.0265** | 2.0659** | 1.5188** | Conserved hypothetical protein |
| IIA/chromosome | RCFBP_11871 | 1 | 0.0265** | 1.9795** | 1.5188** | Conserved hypothetical protein |
| IIA/chromosome | RCFBP_20933 - RCFBP_20934 | 1 | 0.0248** | 2.1239** | 1.5085** | Intergenic region |
| IIB/chromosome | RSPO_c00534 | 1 | 0.0183** | 2.1298** | 1.4823** | Hypothetical cytosolic protein |
| IIB/chromosome | RSPO_c01192 | 1 | 0.0324** | 3.0226** | 1.6101** | Conserved hypothetical protein |
| IIB/chromosome | RSPO_c01332 - RSPO_c01333 | 1 | 0.0451** | 2.0604** | 1.4903** | Intergenic region |
| IIB/chromosome | RSPO_c02427 | 1 | 0.0183** | 2.0777** | 1.4823** | Conserved hypothetical protein |
| IIB/chromosome | RSPO_c02337 | 1 | 0.0183** | 2.1871** | 1.4823** | Conserved hypothetical protein |
| IIB/chromosome | RSPO_c01923 | 1 | 0.0240** | 2.7797** | 1.5475** | Conserved hypothetical protein |
| IIB/chromosome | RSPO_c02827 | 2 | 0,0352** | 2.0961** | 1.6253** | Conserved hypothetical protein |
| IIB/chromosome | RSPO_c02725 - RSPO_c02726 | 1 | 0.0211** | 2.0978** | 1.5182** | Intergenic region |
| IIB/chromosome | RSPO_c03232 - RSPO_c03231 | 1 | 0.0226** | 2.1389** | 1.5335** | Intergenic region |
| IIB/megaplasmid | RSPO_m00507 - RSPO_m00508 | 1 | 0.0338** | 2.7577** | 1.3908** | Intergenic region |
| IIB/megaplasmid | RSPO_m00342 - RSPO_m00343 | 1 | 0.0282** | 2.1609** | 1.5826** | Intergenic region |
| IIB/megaplasmid | RSPO_m01597 | 3 | 0.0465** | 2.7299** | 1.6703** | Conserved hypothetical protein |
| IV/chromosome | RPSI07_0472 - RPSI07_0473 | 1 | 0.0264** | 1.7003** | 1.7993** | Intergenic region |
| IV/megaplasmid | RPSI07_mp0104 -RPSI07_mp0105 | 1 | 0.1464** | 1.7880** | 1.7880** | Intergenic region |

*^a^* Systematic gene identifier according to GMI1000, CFBP2957, Po82 or PSI07 strain nomenclature for phylotype I, IIA, IIB or IV respectively.

*^b^* Number of significant windows overlapping described gene.

*^c^* Observed values of statistics for each gene and significance of coalescent simulations using standard neutral model: * *p* < 0.1 and ** *p* < 0.05

**Supplementary Table 4.** Sequence identifiers of genomic data used in this study.

| **Strain** | **Replicons** | |  |
| --- | --- | --- | --- |
| GMI1000 | chromosome:NC_003295.1/AL646052.1 | plasmid:NC_003296.1/AL646053.1 | |
| Po82 | chromosome:NC_017574.1/CP002819.1 | plasmid:NC_017575.1/CP002820.1 | |
| PSI07 | chromosome:NC_014311.1/FP885906.2 | plasmid:NC_014310.1/FP885891.2 | |
| UY031 | chromosome:NZ_CP012687.1/CP012687.1 | plasmid:NZ_CP012688.1/CP012688.1 | |
| KACC 10722 | chromosome:NZ_CP014702.1/CP014702.1 | plasmid:NZ_CP014703.1/CP014703.1 | |
| UW163 | chromosome:NZ_CP012939.1/CP012939.1 | plasmid:NZ_CP012941.1/CP012941.1 | |
| IBSBF1503 | chromosome:NZ_CP012943.1/CP012943.1 | plasmid:NZ_CP012944.1/CP012944.1 | |
| KACC10709 | chromosome:NZ_CP016904.1/CP016904.1 | plasmid:NZ_CP016905.1/CP016905.1 | |
| OE1-1 | chromosome:NZ_CP009764.1/CP009764.1 | plasmid:NZ_CP009763.1/CP009763.1 | |
| FJAT-1458 | chromosome:NZ_CP016554.1/CP016554.1 | plasmid:NZ_CP016555.1/CP016555.1 | |
| FJAT-91 | chromosome:NZ_CP016612.1/CP016612.1 | plasmid:NZ_CP016613.1/CP016613.1 | |
| SEPPX05 | chromosome:NZ_CP021448.1/CP021448.1 | plasmid:NZ_CP021449.1/CP021449.1 | |
| CQPS-1 | chromosome:NZ_CP016914.1/CP016914.1 | plasmid:NZ_CP016915.1/CP016915.1 | |
| RS 488 | chromosome:NZ_CP021652.1/CP021652.1 | plasmid:NZ_CP021653.1/CP021653.1 | |
| RS 489 | chromosome:NZ_CP021766.1/CP021766.1 | plasmid:NZ_CP021767.1/CP021767.1 | |
| FQY_4 | chromosome:NC_020799.1/CP004012.1 | plasmid:NC_021745.1/CP004013.1 | |
| Rs-10-244 | chromosome:NZ_CM002755.1/CM002755.1 | plasmid:NZ_CM002756.1/CM002756.1 | |
| Rs-09-161 | chromosome:NZ_CM002757.1/CM002757.1 | plasmid:NZ_CM002758.1/CM002758.1 | |
| YC45 | chromosome:CP011997.1 | plasmid:CP011998.1 | |
| IPO1609 | NZ_CDGL000000000.1 |  | |
| RD15 | NZ_MNCM00000000.1 |  | |
| PSS4 | NZ_MOLK00000000.1 |  | |
| GEO_96 | NZ_MZNA00000000.1 |  | |
| SD54 | NZ_ASQR00000000.2 |  | |
| 23-10BR | NZ_JQOI00000000.1 |  | |
| CIP120 | NZ_JXAY00000000.1 |  | |
| P597 | NZ_JIBY00000000.1 |  | |
| CFBP6783 | NZ_JXAZ00000000.1 |  | |
| UW491 | NZ_LVKU00000000.1 |  | |
| UW24 | NZ_LVKT00000000.1 |  | |
| UW365 | NZ_LVKS00000000.1 |  | |
| UW551 | NZ_LVKV00000000.1 |  | |
| PSS1308 | NZ_MOLO00000000.1 |  | |
| PSS190 | NZ_MOLO00000000.1 |  | |
| UW25 | NZ_NCTK00000000.1 |  | |
| GEO_304 | NZ_PHHO00000000.1 |  | |
| B50 | NZ_NCTK00000000.1 |  | |
| UW179 | NZ_CDLZ00000000.1 |  | |
| CIP417 | NZ_CDLZ00000000.1 |  | |
| Grenada 9-1 | NZ_CDLW00000000.1 |  | |
| CFBP1416 | NZ_CDLX00000000.1 |  | |
| CFBP7014 | NZ_CDRJ00000000.1 |  | |
| IBSBF1900 | NZ_CDRW00000000.1 |  | |
| RS2 | NZ_CDRX00000000.1 |  | |
| UW181 | NZ_CDSB00000000.1 |  | |
| CFBP3858 | NZ_CDQJ00000000.1 |  | |
| Rs-T02 | NZ_LKVH00000000.1 |  | |
| UW757 | NZ_LFJP00000000.1 |  | |
| BBAC-C1 | NZ_MKKZ00000000.1 |  | |
| CFBP2957 | FP885897.1 |  | |
| K60 | CAGT00000000.1 |  | |
| UW700 | NCTJ00000000.1 |  | |
| RD13-01 | LN899822 |  | |
| TO10 | LN899827 |  | |
| A2-HR MARDI | NZ_CP019911.1 |  | |
| R229 | FR854059 to FR854085 |  | |
| R24 | FR854086 to FR854092 |  | |

**Supplementary Table 5.** Estimation of nucleotide diversity and nonsynonymous to synonymous substitution rate ratio of RSSC genes under BS.

| **Phylotype/replicon** | **Gene ID*^a^*** | **Nucleotide diversity (π)** | **Gene sites with positive selection*^a^*** |
| --- | --- | --- | --- |
| I/chromosome | RSc2735 | 0.05316 | 1 |
| I/chromosome | RSc2736 | 0.03842 | 3 |
| I/chromosome | RSc0688 | 0.00748 | 1 |
| I/chromosome | RSc2066 | 0.10757 | 2 |
| I/chromosome | RSc2670 | 0.01730 | 0 |
| I/chromosome | RSc2669 | 0.02698 | 1 |
| I/megaplasmid | RSp0832 | 0.01174 | 7 |
| I/megaplasmid | RSp0304 | 0.02680 | 11 |
| I/megaplasmid | RSp0487 | 0.01062 | 2 |
| I/megaplasmid | RSp1212 | 0.00086 | 1 |
| I/megaplasmid | RSp0238 | 0.00774 | 3 |
| I/megaplasmid | RSp1530 | 0.00355 | 3 |
| I/megaplasmid | RSp1100 | 0.01026 | 3 |
| IIA/chromosome | RCFBP_11371 | 0.01750 | 5 |
| IIA/chromosome | RCFBP_11349 | 0.01420 | 0 |
| IIA/chromosome | RCFBP_20503 | 0.01025 | 0 |
| IIA/chromosome | RCFBP_11056 | 0.01654 | 1 |
| IIA/chromosome | RCFBP_10967 | 0.01367 | 2 |
| IIA/chromosome | RCFBP_21311 | 0.01091 | 2 |
| IIA/chromosome | RCFBP_10305 | 0.02164 | 1 |
| IIA/chromosome | RCFBP_10218 | 0.02431 | 3 |
| IIA/chromosome | RCFBP_11858 | 0.01293 | 1 |
| IIA/chromosome | RCFBP_10092 | 0.01633 | 1 |
| IIA/chromosome | RCFBP_10712 | 0.00981 | 0 |
| IIA/chromosome | RCFBP_10711 | 0.00684 | 3 |
| IIA/chromosome | RCFBP_21242 | 0.00891 | 1 |
| IIA/chromosome | RCFBP_20936 | 0.02073 | 1 |
| IIA/chromosome | RCFBP_10686 | 0.06466 | 7 |
| IIA/chromosome | RCFBP_11806 | 0.05515 | 15 |
| IIA/chromosome | RCFBP_11870 | 0.02218 | 9 |
| IIA/chromosome | RCFBP_20594 | 0.04513 | 29 |
| IIA/megaplasmid | RCFBP_mp10317 | 0.00893 | 1 |
| IIA/megaplasmid | RCFBP_mp10609 | 0.04097 | 24 |
| IIA/megaplasmid | RCFBP_mp30035 | 0.00801 | 1 |
| IIA/megaplasmid | RCFBP_mp30119 | 0.02985 | 4 |
| IIA/megaplasmid | RCFBP_mp30438 | 0.01814 | 5 |
| IIA/megaplasmid | RCFBP_mp20003 | 0.06078 | 5 |
| IIB/chromosome | RSPO_c00124 | 0.00424 | 0 |
| IIB/chromosome | RSPO_c00113 | 0.01196 | 0 |
| IIB/chromosome | RSPO_c00179 | 0.01131 | 12 |
| IIB/chromosome | RSPO_c00415 | 0.02156 | 3 |
| IIB/chromosome | RSPO_c00497 | 0.01247 | 0 |
| IIB/chromosome | RSPO_c00765 | 0.01171 | 1 |
| IIB/chromosome | RSPO_c02646 | 0.02601 | 7 |
| IIB/chromosome | RSPO_c01209 | 0.01354 | 2 |
| IIB/chromosome | RSPO_c01332 | 0.02288 | 4 |
| IIB/chromosome | RSPO_c02391 | 0.02940 | 13 |
| IIB/chromosome | RSPO_c02306 | 0.02145 | 2 |
| IIB/chromosome | RSPO_c01998 | 0.06631 | 28 |
| IIB/chromosome | RSPO_c01999 | 0.03369 | 44 |
| IIB/chromosome | RSPO_c01798 | 0.02825 | 26 |
| IIB/chromosome | RSPO_c01795 | 0.00824 | 0 |
| IIB/chromosome | RSPO_c00909 | 0.02391 | 1 |
| IIB/chromosome | RSPO_c01066 | 0.00824 | 0 |
| IIB/chromosome | RSPO_c01082 | 0.01631 | 3 |
| IIB/chromosome | RSPO_c03170 | 0.01714 | 3 |
| IIB/chromosome | RSPO_c03029 | 0.01468 | 5 |
| IIB/megaplasmid | RSPO_m01227 | 0.01920 | 3 |
| IIB/megaplasmid | RSPO_m01150 | 0.01479 | 0 |
| IIB/megaplasmid | RSPO_m00202 | 0.12444 | 15 |
| IIB/megaplasmid | RSPO_m00035 | 0.03842 | 22 |
| IIB/megaplasmid | RSPO_m01206 | 0.03904 | 8 |
| IIB/megaplasmid | RSPO_m01229 | 0.03127 | 37 |
| IIB/megaplasmid | RSPO_m01312 | 0.02707 | 24 |
| IIB/megaplasmid | RSPO_m01371 | 0.03244 | 16 |
| IIB/megaplasmid | RSPO_m00869 | 0.04060 | 14 |
| IIB/megaplasmid | RSPO_m00770 | 0.03500 | 16 |
| IIB/megaplasmid | RSPO_m01600 | 0.07808 | 116 |
| IIB/megaplasmid | RSPO_m01541 | 0.00951 | 1 |
| IV/chromosome | RPSI07_1784 | 0.01716 | 0 |
| IV/chromosome | RPSI07_2871 | 0.02017 | 3 |
| IV/chromosome | RPSI07_1208 | 0.02271 | 0 |
| IV/chromosome | RPSI07_1185 | 0.03402 | 1 |
| IV/chromosome | RPSI07_0660 | 0.00994 | 0 |
| IV/chromosome | RPSI07_0072 | 0.11380 | 8 |
| IV/chromosome | RPSI07_0735 | 0.08009 | 4 |
| IV/megaplasmid | RPSI07_mp0105 | 0.01037 | 0 |
| IV/megaplasmid | RPSI07_mp0022 | 0.01140 | 2 |
| Average |  | 0.027 | 7.7 |

***^a^*** Number of sites in the respective gene under positive selection calculated with the mixed-effects model of evolution method and *p*-value at 0.25.

**Supplementary Figure 1.** Observed versus simulated values of summary statistics for Phylotype I/chromosome. SNM, PCM and BNM indicate simulations under three different demographic scenarios: standard neutral model, population contraction model and bottleneck model, respectively. Asterisk indicates significant *p*-values (0.05) for the respective comparisons.

*****

*****

*****

*****

*****

*****

*****

*****

*****

*****

*****

*****

*****

*****

*****

***v**

*****

*****

*****

*****

*****

*****

*****

*****

*****

*****

*****

*****

*****

*****

*****

*****

*****

*****

*****

*****

*****

*****
